# Developmental Timing of Alcohol Initiation as a Marker of Adult Clinical Complexity in Alcohol Use Disorder: A Cross-Sectional Study

**DOI:** 10.3390/medsci14050535

**Published:** 2026-08-31

**Authors:** María Flores-López, Raquel Reviriego, Nuria García-Marchena, Nerea Requena-Ocaña, Juan Jesús Ruiz-Ruiz, Jesús Herrera-Imbroda, Antonio Bordallo, Juan Suarez, Patricia Moreno-Peral, Juan Ángel Bellón, Fernando Rodríguez de Fonseca, Francisco Javier Pavón-Morón, Antonia Serrano

**Affiliations:** 1 IBIMA Plataforma BIONAND, Instituto de Investigación Biomédica de Málaga, 29590 Málaga, Spain; maria.flores@ibima.eu (M.F.-L.); raquel.reviriego@ibima.eu (R.R.); nereareq@uma.es (N.R.-O.); jesus.herrera.imbroda.sspa@juntadeandalucia.es (J.H.-I.); juan.suarez@uma.es (J.S.); patriciamorenoperal@uma.es (P.M.-P.); jabellon@uma.es (J.Á.B.); antonia.serrano@ibima.eu (A.S.); 2 Unidad de Gestión Clínica de Salud Mental, Hospital Regional Universitario de Málaga, 29010 Málaga, Spain; antonio.bordallo.sspa@juntadeandalucia.es; 3 Departamento de Psicobiología, Facultad de Psicología, Universidad Complutense de Madrid, Pozuelo de Alarcón, 28223 Madrid, Spain; ngmarchena@ucm.es; 4 Departamento de Psicobiología y Metodología de las Ciencias del Comportamiento, Universidad de Málaga, 29010 Málaga, Spain; 5 Centro Provincial de Drogodependencias, Diputación Provincial de Málaga, 29010 Málaga, Spain; jjruiz@malaga.es; 6 Departamento de Anatomía Humana, Medicina Legal e Historia de la Ciencia, Universidad de Málaga, 29010 Málaga, Spain; 7 Departamento de Personalidad, Evaluación y Tratamiento Psicológico, Universidad de Málaga, 29010 Málaga, Spain; 8 Research Network on Chronicity, Primary Care, Prevention and Health Promotion (RICAPPS), Instituto de Salud Carlos III, 28029 Madrid, Spain; 9 Centro de Salud El Palo, Servicio Andaluz de Salud, 29018 Málaga, Spain; 10 Departamento de Salud Pública y Psiquiatría, Universidad de Málaga, 29010 Málaga, Spain; 11 Unidad de Gestión Clínica de Neurología, Hospital Regional Universitario de Málaga, 29010 Málaga, Spain; 12 Área del Corazón, Hospital Universitario Virgen de la Victoria, 29010 Málaga, Spain; 13 CIBER de Enfermedades Cardiovasculares (CIBERCV), Instituto de Salud Carlos III, 28029 Madrid, Spain

**Keywords:** alcohol use disorder, adolescence, age at alcohol initiation, polysubstance use, externalizing psychopathology, clinical phenotype, treatment-seeking population

## Abstract

**Background/Objectives**: Age at alcohol initiation is consistently associated with later alcohol use disorder (AUD), but it remains uncertain whether initiation across distinct developmental stages identifies clinically different adult phenotypes. We examined whether alcohol initiation during early, middle, or late adolescence was associated with graded differences in addictive, psychiatric, and treatment-related complexity among adults with AUD. **Methods**: This cross-sectional study included 436 treatment-seeking adults with lifetime AUD recruited from addiction programs in Spain. Primary analyses included 409 participants who reported alcohol initiation during early (10–13 years; *n* = 85), middle (14–16 years; *n* = 182), or late adolescence (17–21 years; *n* = 142). Participants underwent structured assessment with the Psychiatric Research Interview for Substance and Mental Disorders (PRISM). Between-group comparisons, ordinal logistic regression, and exploratory latent class analysis (LCA) were performed. **Results**: Earlier initiation was associated with graded increases in comorbid non-alcohol substance use disorders (67.1%, 64.8%, and 47.2% across early, middle, and late initiation; FDR-adjusted *p* = 0.003), childhood conduct disorder (21.2%, 9.3%, and 4.2%; FDR-adjusted *p* < 0.001), and higher rates of lifetime therapeutic community treatment and mutual-support participation. Median age at AUD diagnosis was 19.0, 23.5, and 30.5 years, respectively (*p* < 0.001). In the model including age at AUD diagnosis, childhood conduct disorder (OR = 3.42, 95%CI 1.50–7.76) and mood disorder (OR = 2.13, 95%CI 1.41–3.21) were associated with earlier initiation. LCA identified a high externalizing/polysubstance complexity class (15.2%) enriched for earlier initiation (*p* < 0.001). **Conclusions**: Developmental timing of alcohol initiation was associated with a multidomain adult AUD phenotype. These findings suggest that initiation age could serve as a simple historical prompt for broader assessment; however, its incremental clinical utility was not tested, and it should not be interpreted as evidence of causality or a validated prediction tool.

## 1. Introduction

Alcohol is a psychoactive, toxic, and dependence-producing substance that remains a major global public health concern. The latest World Health Organization report estimated that alcohol use accounted for approximately 2.6 million deaths (4.7% of all deaths) and 115.9 million disability-adjusted life years worldwide in 2019 [1]. Europe continues to have the highest levels of alcohol consumption globally, with a substantial burden of preventable disease and injury [2]. In Spain, 92.9% of people aged 15–64 years reported lifetime alcohol use in the 2025 national survey [3]. Beyond its direct toxic effects, alcohol use is associated with broad medical and psychiatric morbidity that increases clinical complexity and health care needs [4,5].

A younger age at first alcohol use has repeatedly been associated with greater risk of later AUD, more severe alcohol-related problems, and involvement with other substances [6,7,8,9]. Recent longitudinal studies continue to support associations between earlier or riskier adolescent drinking and adverse substance-use outcomes in young adulthood [10,11]. However, the clinically relevant question is no longer simply whether “early onset” matters. A more precise issue is whether the developmental stage during which alcohol use begins helps distinguish adult AUD phenotypes among people who ultimately enter treatment.

Adolescence is not a homogeneous developmental period. Early, middle, and late adolescence differ in neurobiological maturation, emotional regulation, social context, and exposure to peer and environmental influences [12,13]. Prefrontal–limbic systems involved in reward processing, inhibitory control, decision-making, and emotion regulation continue to mature throughout adolescence [14,15], while identity formation, autonomy, and risk-taking also change across this interval [16]. Accordingly, alcohol initiation during the earliest developmental window may mark a different constellation of vulnerability than initiation closer to adulthood. The exact boundaries of these stages vary across frameworks; therefore, the present categories should be understood as prespecified, developmentally informed strata rather than universally accepted biological cutoffs.

The association between early alcohol initiation and later psychopathology is also bidirectional and confounded by shared liability. Alcohol exposure during adolescence may be related to neurodevelopmental changes, whereas pre-existing externalizing traits, affective symptoms, family influences, and environmental adversity may facilitate earlier initiation [17,18,19,20,21]. Twin and genetically informed studies further caution that early initiation can function as an indicator of shared vulnerability rather than an isolated causal exposure [22]. For this reason, age at initiation is best evaluated as a clinically informative marker that co-occurs with addictive, externalizing, affective, and social complexity.

Most available evidence comes from general-population cohorts, which characterize drinking milestones well but often lack the structured diagnostic depth required to define adult clinical phenotypes. The contribution of the present study is therefore not to re-establish that earlier drinking is associated with later alcohol problems, but to test whether prespecified early-, middle-, and late-adolescent initiation stages distinguish multidomain phenotypes among adults who already have AUD. Using a large treatment-seeking clinical cohort assessed with the Psychiatric Research Interview for Substance and Mental Disorders (PRISM) [23,24], we integrated false discovery rate (FDR)-controlled ordered trends, adjusted ordinal regression, and an exploratory latent class analysis (LCA) constructed without initiation stage. We hypothesized that earlier initiation would be associated with graded increases in polysubstance involvement, addiction severity, psychiatric and medical comorbidity, and lifetime treatment exposure, particularly externalizing and affective psychopathology. All analyses were interpreted as cross-sectional phenotype associations rather than evidence of causal pathways or incremental clinical utility.

## 2. Materials and Methods

### 2.1. Study Design, Participants, and Recruitment

This multicenter cross-sectional study included 436 adults with a lifetime diagnosis of AUD according to the Diagnostic and Statistical Manual of Mental Disorders, Fifth Edition (DSM-5) [25]. Participants were consecutively recruited between January 2021 and December 2025 from outpatient treatment programs specializing in addictions and substance use disorders (SUDs) throughout the province of Málaga, Spain, including the Provincial Center for Drug Dependence (Centro Provincial de Drogodependencias [CPD]) and the participating outpatient treatment centers. The same recruitment window was applied across all participating centers.

Eligibility criteria were age 18 years or older, a lifetime history of AUD, and abstinence from substance use at the time of assessment. Exclusion criteria were inability to complete the psychopathological or cognitive assessment and insufficient fluency in Spanish. Alcohol initiation was defined as the age at which the participant consumed their first full standard alcoholic drink, rather than merely a sip or taste.

At the study assessment, abstinence was ascertained by self-report and supported by the toxicological monitoring routinely conducted as part of the outpatient treatment programs. At admission to the treatment program, participants underwent blood and urine testing. Thereafter, substance use was routinely monitored through toxicological testing every 15 days and additionally whenever substance use was suspected. These procedures were not standardized study-specific laboratory assessments, and their results were used only to support the clinical ascertainment of abstinence rather than as study outcomes. No predefined minimum abstinence interval before the study assessment was required.

For the primary stage-based analyses, participants reporting initiation between 10 and 21 years were classified into three prespecified, developmentally informed groups: early adolescence (10–13 years), middle adolescence (14–16 years), and late adolescence/transition to adulthood (17–21 years) [12,13]. Participants initiating after 21 years were described as the adult-initiation group but were excluded from the primary comparisons because of the small group size (*n* = 27).

### 2.2. Ethics

All participants provided written informed consent after receiving a detailed explanation of the study procedures and having the opportunity to ask questions. The data included in the present analysis were collected under two research protocols that were approved by the Research Ethics Committee of Málaga (Portal de Ética de la Investigación Biomédica de Andalucía [PEIBA], Regional Government of Andalusia; protocol codes PI20_01399-2020 and PI22_00427-2022, approved on 27 November 2020, and 30 March 2023, respectively). This study was conducted in accordance with the Declaration of Helsinki and applicable Spanish and European data-protection regulations, including Regulation (EU) 2016/679. Study data were pseudonymized using unique study identifiers before analysis.

### 2.3. Clinical Assessments

Assessments were conducted individually by trained and experienced psychologists in a quiet, private setting and lasted approximately 120–140 min. The validated Spanish-language PRISM, adapted to apply DSM-5 diagnostic criteria, was used to assess substance-specific use histories, SUD diagnoses and severity, and co-occurring psychiatric disorders [23,24]. The interview assessed both lifetime and current diagnoses and distinguished independent psychiatric disorders from substance-induced disorders. The PRISM incorporates a consumption-based life chart to anchor the reported onset and course of substance use, periods of abstinence, and diagnostic milestones. The psychiatric domains assessed included mood, anxiety, psychotic, personality, and attention-deficit/hyperactivity disorders (ADHD). Sociodemographic, medical, legal, treatment-history, and mutual-support variables were also recorded. Clinical information, including medical comorbidities and treatment history, was obtained from both participant interviews and available medical records. Treatment-related variables were operationalized as lifetime treatment/support setting (i.e., none, outpatient care, hospitalization, or both), therapeutic community treatment, and mutual-support group participation. These indicators capture treatment exposure or engagement; they do not quantify treatment dose, session frequency, duration, need, effectiveness, or response.

### 2.4. Statistical Analysis

Continuous variables were evaluated for distributional assumptions and are summarized as median and interquartile range (IQR); categorical variables are reported as number and percentage. The overall cohort was first described according to sociodemographic characteristics, substance use patterns, and AUD-related variables. Primary comparative analyses were restricted to the 409 participants who initiated alcohol use between 10 and 21 years.

Categorical variables were compared with the chi-square test or Fisher’s exact test, as appropriate, and continuous or ordinal variables with the Kruskal–Wallis test. Because the initiation-stage variable was ordered, monotonic gradients across early, middle, and late initiation were additionally evaluated. Jonckheere–Terpstra tests were used for continuous or ordinal outcomes, and Cochran–Armitage trend tests were used for binary outcomes. The FDR was controlled with the Benjamini–Hochberg procedure for the prespecified main trend analyses [26].

Ordinal logistic regression was used to identify variables associated with an earlier initiation stage. The outcome was coded so that an odds ratio (OR) greater than 1 represented higher odds of belonging to an earlier stage. Candidate predictors were current age, sex, criminal record, number of SUD diagnoses, cocaine use disorder, cannabis use disorder, number of AUD severity criteria, mood disorder, childhood conduct disorder, and antisocial personality disorder. Model 1 included age at AUD diagnosis; Model 2 excluded it because of its conceptual and temporal proximity to age at alcohol initiation. ORs are reported with 95% confidence intervals (CIs), and the proportional-odds assumption was evaluated with the test of parallel lines.

An exploratory LCA based on Bernoulli mixture modeling was used to identify clinical subgroups from binary indicators of criminal record, cocaine use disorder, cannabis use disorder, mood disorder, childhood conduct disorder, antisocial personality disorder, ADHD, therapeutic community treatment, and mutual-support group participation. The composite “comorbid non-alcohol SUD” variable was excluded to avoid redundancy. One- through five-class solutions were compared using the Akaike information criterion, Bayesian information criterion (BIC), entropy, minimum class size, and clinical interpretability. Initiation stage was not used to construct the classes and was compared across classes only after model selection. Of the 409 participants included in the primary stage-based analyses, one had missing data for at least one of the LCA indicators and was therefore excluded only from the LCA. The LCA was consequently conducted using 408 complete cases. Two-sided *p*-values < 0.05 were considered statistically significant. Statistical analyses were performed using JASP version 0.98.0 (JASP Team, 2026, Amsterdam, The Netherlands). The LCA was conducted in R version 4.6.1 (R Foundation for Statistical Computing, Vienna, Austria) using the poLCA package (version 1.6.0.2). Figures were generated in Python version 3.12.13 using Matplotlib version 3.10.9. Participants were assigned to their most likely class using maximum posterior probability. Because the fit indices diverged and adjusted BIC favored three classes, the three-class solution was additionally inspected as a sensitivity analysis to determine whether the two-class structure obscured clinically meaningful heterogeneity.

## 3. Results

### 3.1. Overall Cohort Characteristics

The overall cohort included 436 participants, most of whom were men (77.5%); median age was 45.5 years (IQR 36.25–53.0) and median body mass index was 25.30 kg/m^2^ (IQR 22.87–28.68). Unemployment was the most frequent employment status (45.6%), and 33.9% had completed primary education or less. Comorbid non-alcohol SUDs were common (58.5%), particularly cocaine use disorder (47.2%) and cannabis use disorder (20.2%); 70.6% reported nicotine use. Median age at alcohol initiation was 16 years (IQR 14–18), median age at AUD diagnosis was 25 years (IQR 19–34), and median AUD severity was eight criteria (IQR 6–9). Most participants initiated alcohol use between 10 and 21 years (*n* = 409, 93.8%); 27 (6.2%) reported initiation after age 21 (Table 1).

### 3.2. Clinical and Alcohol-Related Characteristics by Initiation Stage

The primary analytic sample comprised 85 participants in the early-adolescence group, 182 in the middle-adolescence group, and 142 in the late-adolescence group. Sex distribution and current age did not differ significantly across groups. A criminal record was more common among participants with early or middle initiation than among those with late initiation (40.0%, 38.5%, and 23.2%, respectively; *p* = 0.006). Earlier initiation was also associated with greater addictive complexity: cannabis use disorder occurred in 29.4%, 24.7%, and 9.9% of the groups (*p* < 0.001), cocaine use disorder in 54.1%, 51.6%, and 38.7% (*p* = 0.028), and any comorbid non-alcohol SUD in 67.1%, 64.8%, and 47.2% (*p* = 0.001), respectively.

The number of SUD diagnoses differed across stages (*p* < 0.001). Median age at AUD diagnosis increased from 19.0 years in the early group to 23.5 years in the middle group and 30.5 years in the late group (*p* < 0.001). Participants with earlier initiation met more AUD severity criteria (*p* = 0.003). Alcohol abstinence duration, tobacco use, and cigarettes per day did not differ significantly (Table 2).

### 3.3. Substance-Specific Profiles

Among participants with cannabis use disorder, cannabis severity criteria were higher in the earlier-initiation groups (median 6.0, 4.0, and 2.0; *p* = 0.013), although age at cannabis initiation and diagnosis did not differ significantly. For cocaine, earlier alcohol initiation was associated with younger cocaine initiation (median 15.0, 18.0, and 22.0 years; *p* < 0.001), younger cocaine use disorder diagnosis (20.0, 23.0, and 28.0 years; *p* < 0.001), and greater cocaine disorder severity (*p* = 0.012). These severity and timing analyses were restricted to participants with the corresponding diagnosis (Table 3).

### 3.4. Psychiatric, Medical, and Treatment-Related Characteristics

Overall psychiatric comorbidity did not differ significantly across initiation stages, but mood disorder was more frequent with earlier initiation (56.5%, 45.1%, and 38.7%; *p* = 0.034). Childhood conduct disorder showed a marked gradient (21.2%, 9.3%, and 4.2%; *p* < 0.001), as did antisocial personality disorder (16.5%, 11.5%, and 4.9%; *p* = 0.016). Anxiety disorders, psychotic disorders, ADHD, and broad personality-disorder categories did not differ significantly (Table S1). Overall medical comorbidity was similar across stages; the observed difference in musculoskeletal illness (*p* = 0.021) involved small counts and should be considered exploratory (Table S2).

Treatment-history differences were more pronounced. The distribution of lifetime psychological or substance-use treatment/support settings (i.e., none, outpatient care, hospitalization, or both) differed by initiation stage (*p* = 0.002). Therapeutic community treatment was reported by 29.4%, 17.6%, and 11.9% of participants in the early, middle, and late groups, respectively (*p* = 0.004), and mutual-support group participation by 50.6%, 28.1%, and 27.5% (*p* < 0.001). These findings indicate greater lifetime treatment exposure or engagement in the earlier-initiation groups; the variables did not measure the frequency, duration, or dose of care and cannot establish treatment need, access, effectiveness, response, or temporal sequence.

A detailed breakdown of the individual psychiatric diagnoses assessed with the PRISM is provided in Table S3.

### 3.5. Ordered Trends Across Developmental Stages

**Figure 1:**
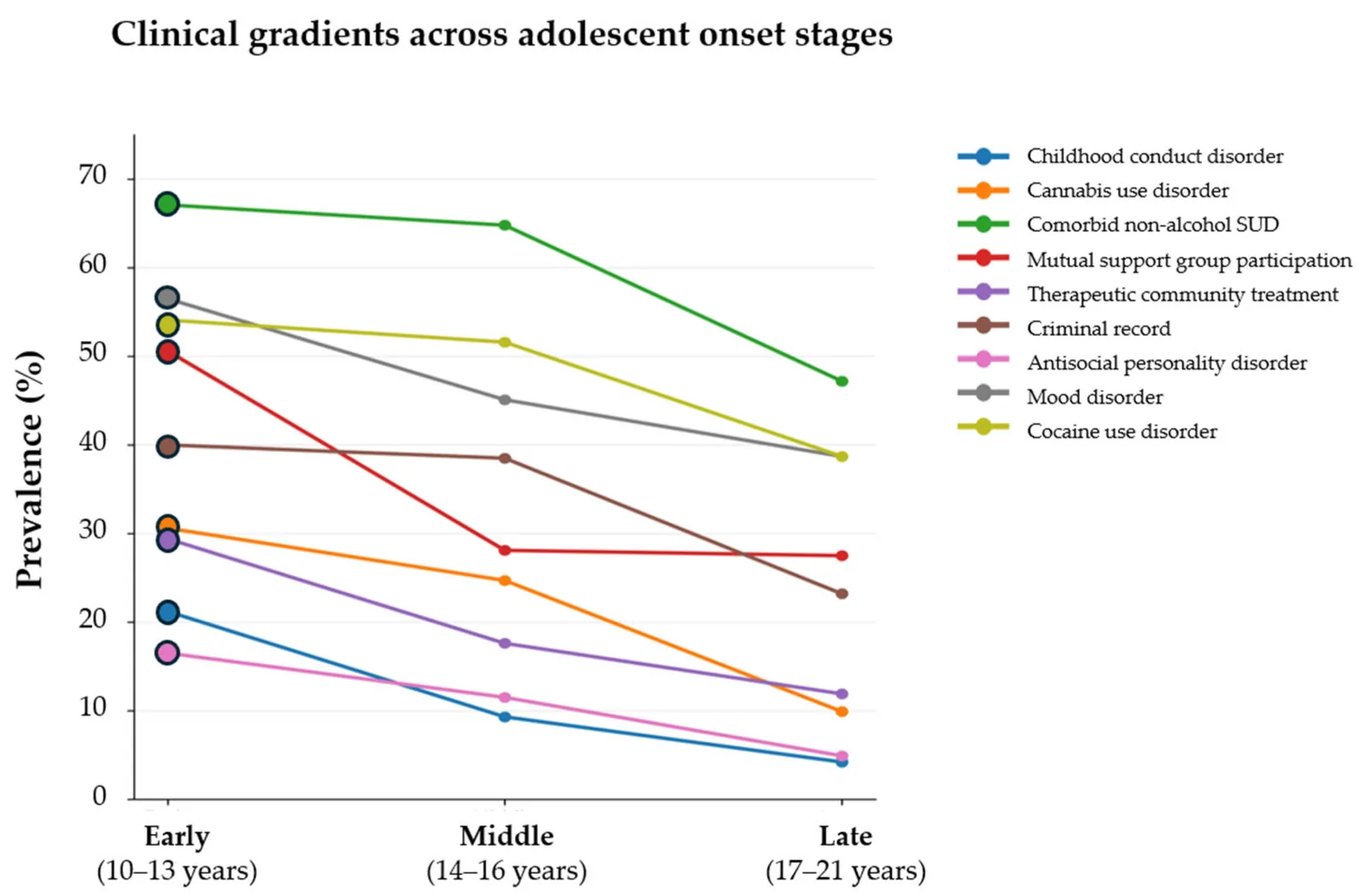
Prevalence of clinical indicators with significant ordered trends across developmental stages of alcohol initiation. Values are observed percentages. Trend *p* values and FDR-adjusted *p* values are reported in Table S4. SUD, substance use disorder.

Nine prespecified binary indicators showed monotonic trends across early, middle, and late initiation that remained significant after FDR correction: childhood conduct disorder, cannabis use disorder, comorbid non-alcohol SUD, mutual-support group participation, therapeutic community treatment, criminal record, antisocial personality disorder, mood disorder, and cocaine use disorder (all FDR-adjusted *p* ≤ 0.017; Table S4). Psychiatric comorbidity (trend *p* = 0.057) and ADHD (trend *p* = 0.108) did not reach statistical significance. These significant gradients are summarized in Figure 1.

### 3.6. Ordinal Logistic Regression

The proportional-odds assumption was reported as supported for the full model. In Model 1, which included age at AUD diagnosis, current age (OR = 1.02, 95% CI 1.00–1.05; *p* = 0.036), childhood conduct disorder (OR = 3.42, 95% CI 1.50–7.76; *p* = 0.003), and mood disorder (OR = 2.13, 95% CI 1.41–3.21; *p* < 0.001) were associated with earlier initiation, whereas older age at AUD diagnosis was associated with later initiation (OR = 0.92, 95% CI 0.90–0.94; *p* < 0.001). In the sensitivity model excluding age at AUD diagnosis, childhood conduct disorder (OR = 2.84, 95% CI 1.26–6.40; *p* = 0.012) and mood disorder (OR = 1.62, 95% CI 1.10–2.40; *p* = 0.016) remained associated with earlier initiation (Table 4; the complete prespecified model for both specifications, including all candidate predictors, model-fit statistics, and the test of parallel lines, is reported in Table S5).

### 3.7. Exploratory Latent Class Analysis

**Figure 2:**
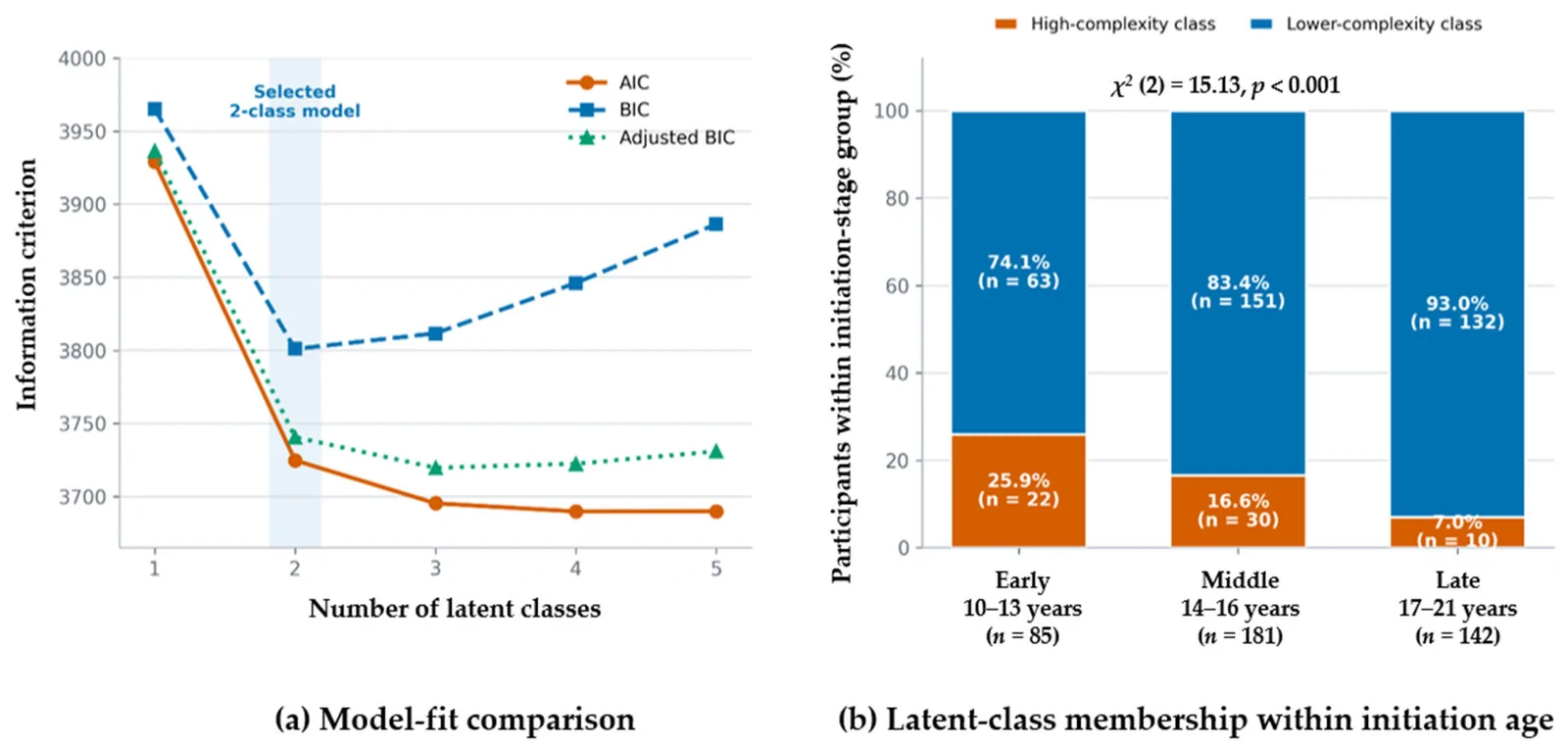
Model-fit comparison and latent-class membership according to developmental stage of alcohol initiation. (**a**) Comparison of the Akaike information criterion (AIC), Bayesian information criterion (BIC), and adjusted BIC across the one- through five-class solutions. Although AIC and adjusted BIC reached their lowest values for the four- and three-class solutions, respectively, the two-class solution was selected based on the lowest BIC (3801.0), the highest entropy among the multi-class solutions (0.811), adequate class size, parsimony, and clinical interpretability. (**b**) Proportions of participants assigned to the high externalizing/polysubstance complexity class and the lower-complexity class within each alcohol-initiation stage. Percentages were calculated separately within each initiation-stage group. High-complexity class membership decreased from 25.9% in the early-initiation group to 16.6% in the middle-initiation group and 7.0% in the late-initiation group. Alcohol-initiation stage was not included as a class-defining indicator. The LCA included 408 participants with complete indicator data, and class membership differed across initiation stages (χ^2^ = 15.13, df = 2, *p* < 0.001). LCA, latent class analysis.

The LCA was conducted among the 408 participants with complete data for all nine class indicators. As shown in Figure 2a and Table S6, the information criteria did not identify the same optimal number of classes: BIC reached its minimum for the two-class solution (3801.0), adjusted BIC for the three-class solution (3719.7), and AIC for the four-class solution (3689.8). This divergence indicates that no single class structure was unequivocally supported; the three-class solution was therefore examined as a plausible sensitivity model. The two-class solution was retained for primary presentation because it had the lowest BIC and the highest entropy among the multi-class solutions (0.811) and provided the most parsimonious and clinically interpretable summary. Its smaller class comprised 62 participants (15.2%).

Class 1, labeled high externalizing/polysubstance complexity, included 62 participants (15.2%) and was characterized by high estimated probabilities of cocaine use disorder (86.4%), criminal record (68.5%), antisocial personality disorder (56.5%), childhood conduct disorder (53.7%), cannabis use disorder (47.7%), and ADHD (47.2%). Class 2, labeled lower complexity, included 346 participants (84.8%) and showed substantially lower estimated probabilities of these indicators.

Latent-class membership differed across alcohol-initiation stages (χ^2^ = 15.13, df = 2, *p* < 0.001; Figure 2b). The proportion of participants assigned to the high externalizing/polysubstance complexity class decreased from 25.9% (22/85) in the early-initiation group to 16.6% (30/181) in the middle-initiation group and 7.0% (10/142) in the late-initiation group. Conversely, the proportions assigned to the lower-complexity class were 74.1% (63/85), 83.4% (151/181), and 93.0% (132/142), respectively. Percentages were calculated separately within each initiation-stage group. Alcohol-initiation stage was not included as a class-defining indicator and was examined only after model selection. The high-complexity class also had a younger median age at AUD diagnosis, greater AUD severity, and more SUD diagnoses.

In the three-class sensitivity solution (AIC = 3695.4, BIC = 3811.7, adjusted BIC = 3719.7, entropy = 0.675; assigned class sizes *n* = 226, 149, and 33), a lower externalizing/polysubstance-complexity class was distinguished from a polysubstance/externalizing class characterized by cocaine use disorder (77.8%), criminal record (49.7%), cannabis use disorder (31.7%), and ADHD (29.7%), and a small marked conduct/antisocial class characterized by antisocial personality disorder (100.0%), childhood conduct disorder (85.5%), cocaine use disorder (82.7%), criminal record (77.3%), ADHD (54.1%), and cannabis use disorder (49.0%). Membership differed across initiation stages (χ^2^ = 25.47, df = 4, *p* < 0.001). The marked conduct/antisocial class decreased from 15.3% of early initiators to 8.3% of middle and 3.5% of late initiators; the corresponding proportions for the polysubstance/externalizing class were 43.5%, 41.4%, and 26.1%. Thus, the three-class solution preserved the association between earlier initiation and greater clinical complexity but split the more complex group into two sub-profiles. Its lower entropy and very small marked conduct/antisocial class supported retaining the more stable and parsimonious two-class solution for primary presentation. All LCA solutions remain exploratory and require independent replication.

## 4. Discussion

In this treatment-seeking clinical cohort, the developmental timing of alcohol initiation was associated with a graded, multidomain pattern of adult AUD complexity. Earlier initiation co-occurred with greater polysubstance involvement, younger age at AUD diagnosis, somewhat greater AUD severity, externalizing and affective psychopathology, and greater lifetime treatment exposure or engagement. Childhood conduct disorder and mood disorder remained associated with earlier initiation in adjusted ordinal models, and the data-driven two-class solution placed a high externalizing/polysubstance profile disproportionately among early and middle initiators. The alternative three-class solution preserved this gradient while revealing heterogeneity within the more complex group. The convergence of ordered trends, regression, and LCA supports the clinical relevance of initiation timing while underscoring that the findings are associative and cross-sectional and that the exact latent structure remains uncertain.

The principal contribution is not another demonstration of the well-established association between a younger first drink and subsequent alcohol-related problems. Rather, it is the stage-specific, multidomain characterization of adults who already have AUD using a structured, substance-specific psychiatric interview. Treating adolescence as three ordered developmental strata instead of a single early-versus-late dichotomy showed that the middle-adolescent group was often intermediate between the early and late groups across addictive, externalizing, affective, legal, and treatment-history domains. The convergence of FDR-controlled ordered trends and adjusted regression, together with an LCA constructed without initiation stage, provides a non-circular data-driven check on the phenotype gradient. These features add clinical resolution within a treatment-seeking AUD population, but they do not establish causality, prediction, incremental assessment value, or a validated new subtype.

Polysubstance involvement was the most consistent component of the earlier-initiation phenotype. Cannabis and cocaine use disorders, the number of SUD diagnoses, and substance-specific severity generally increased as alcohol initiation moved earlier. This gradient is consistent with recent longitudinal work linking earlier adolescent drinking to broader substance involvement in young adulthood [10,11]. Developmental neuroimmune, reward, and control systems may contribute to this clustering [27,28,29,30], but the present data cannot distinguish effects of alcohol exposure from shared genetic, behavioral, family, and environmental liability. The marker interpretation is therefore more defensible than a direct causal interpretation [17,22].

Externalizing psychopathology formed a second prominent domain. Childhood conduct disorder and antisocial personality disorder showed clear stage gradients, and childhood conduct disorder had the largest adjusted association with earlier initiation. This pattern is consistent with developmental models in which early substance use, conduct problems, impulsivity, sensation seeking, and behavioral disinhibition share partially overlapping liability [31,32,33,34]. Clinically, a history of very early alcohol use should therefore prompt assessment of developmental conduct symptoms, legal problems, impulsive behavior, and other SUDs rather than being treated as an isolated alcohol milestone.

Mood disorder was also associated with earlier initiation and remained significant after adjustment. Alcohol use and affective psychopathology are linked bidirectionally: emotional distress may precede alcohol use as maladaptive coping, while repeated exposure may be associated with later dysregulation [35,36,37]. The absence of clear gradients for anxiety, psychotic, ADHD, and broad personality-disorder categories suggests that initiation timing was not simply a proxy for psychiatric comorbidity in general. Instead, the pattern appeared more specific to externalizing and affective domains [38,39].

Earlier initiation was associated with therapeutic community treatment, combined outpatient and inpatient treatment, and mutual-support participation. These findings are compatible with greater lifetime illness complexity, but treatment variables are influenced by service availability, referral pathways, duration of illness, social support, and patient preferences [40]. They should not be interpreted as direct measures of severity or treatment response. The similar abstinence duration across stages, despite differences in prior treatment exposure, is clinically interesting but cannot establish relapse vulnerability in a cross-sectional design.

The exploratory LCA provided convergent evidence of heterogeneity within AUD, but not a uniquely established class structure. The retained two-class solution summarized a small high externalizing/polysubstance group, described in previous latent-structure studies [41,42,43], and a larger lower-complexity group. The plausible three-class solution preserved the association with initiation stage while separating the more complex portion into a broader polysubstance/externalizing profile and a small marked conduct/antisocial profile. Thus, the substantive conclusion, greater representation of complex profiles among earlier initiators, was consistent across these alternatives, whereas the exact number and boundaries of classes were not. Because initiation stage was excluded from class construction, this convergence is non-circular; however, the lower entropy and small class in the three-class model underscore the need for independent replication before either structure is considered stable or suitable for clinical application.

### 4.1. Clinical and Public Health Relevance

Age at alcohol initiation is inexpensive and easy to collect, but the present study did not test its incremental clinical utility because all participants had already entered treatment and completed the comprehensive PRISM assessment. A potential use in routine care would be as a low-burden intake prompt when a full structured interview is not immediately feasible: recording the age at the first full standard drink, particularly initiation at 10–13 years, could remind clinicians to ensure assessment of cocaine and cannabis disorders, developmental conduct and antisocial features, mood disorders, legal problems, and prior treatment/support exposure. It should not by itself establish a diagnosis, label risk, determine treatment allocation, or imply causality. Prospective implementation studies should compare otherwise similar workflows with and without this marker and test incremental assessment yield, referral decisions, and patient outcomes before formal adoption. At the population level, the findings remain compatible with prevention strategies that delay alcohol initiation while emphasizing the externalizing, affective, family, and social vulnerabilities that may precede early drinking.

### 4.2. Strengths and Limitations

Strengths include the relatively large treatment-seeking cohort, substance-specific and psychiatric characterization with the PRISM, analysis of adolescence as an ordered developmental period rather than a single dichotomy, FDR correction for main trend analyses, and convergence across conventional and exploratory analytic approaches.

Several limitations are important. First, the cross-sectional design precludes causal inference and does not resolve the temporal ordering of initiation, psychopathology, polysubstance use, and treatment exposure. Second, initiation age and other milestones were reported retrospectively, often decades after the events, and may be affected by forgetting, rounding, or forward or backward telescoping. The PRISM consumption-based life chart provides temporal anchors but does not independently validate first-drink age. Categorization may reduce reliance on exact single-year recall, yet participants near cut points could be assigned to an adjacent stage; non-differential or differential misclassification could attenuate or distort the observed gradients. Third, initiation was categorized using prespecified age ranges whose boundaries vary across developmental frameworks; the 17–21-year group includes the transition to adulthood. Fourth, the adult-initiation group was too small for stable primary comparisons, limiting conclusions beyond age 21. Fifth, key developmental confounders were not measured or not available for the present analysis, including family history of AUD and other psychiatric disorders, parental substance use, genetic liability, childhood trauma, socioeconomic adversity, peer context, parental monitoring, educational functioning, and neighborhood factors. Residual confounding may therefore account for part of the observed gradients. Sixth, consecutive recruitment within participating centers does not eliminate selection into specialized care. The cohort comprised treatment-seeking adults from outpatient addiction services in Málaga, Spain, who were abstinent at assessment, sufficiently fluent in Spanish, and able to complete a 120–140-min interview. Treatment entry, referral pathways, service availability, and the Spanish health care and legal context may be related to both initiation history and the adult legal and treatment indicators examined. Accordingly, the sample may overrepresent more persistent, complex, or treatment-engaged AUD, and selection could affect both prevalence estimates and within-cohort associations; its direction cannot be determined. Generalizability to women, community samples, younger cohorts, other countries and health systems, and people who never seek care is therefore limited. Seventh, men comprised 77.5% of the cohort. Sex distribution did not differ across initiation stages (*p* = 0.315), and female sex was not independently associated with initiation stage in either adjusted model (Table S5). However, only 13 women were included in the early-initiation group, precluding reliable sex-stratified or interaction analyses. The absence of a sex main effect does not exclude sex-specific effect modification, and the latent profiles may largely reflect the male-dominant sample. Eighth, less common psychiatric and medical analyses were based on small cell counts, and the medical findings should be considered exploratory. Finally, the LCA used complete cases, the fit indices diverged, and the three-class sensitivity model contained a small 33-person class with lower entropy. The exact number and boundaries of classes are therefore uncertain, the two-class solution was not externally validated, and replication in independent and more sex-balanced samples remains essential.

## 5. Conclusions

Among treatment-seeking adults with lifetime AUD, earlier developmental timing of alcohol initiation was associated with a more complex adult phenotype characterized by greater polysubstance involvement, younger age at AUD diagnosis, externalizing and mood pathology, and greater lifetime treatment exposure or engagement. Childhood conduct disorder and mood disorder remained associated with earlier initiation after adjustment. An exploratory two-class solution and a plausible three-class sensitivity solution both placed more complex profiles disproportionately among earlier initiators, although the exact latent structure remains uncertain. Recording initiation age may be a low-burden prompt for comprehensive assessment, but its incremental clinical utility was not tested. It should be viewed as a marker of multidomain vulnerability, not as an isolated causal exposure or a validated risk-stratification tool, until replicated prospectively in independent and more diverse cohorts.

## Figures and Tables

**Table 1:** Sociodemographic characteristics and substance use patterns in the overall AUD cohort.

Domain	Characteristic	AUD Cohort (*n* = 436)
Sex, *n* (%)	Men	338 (77.5)
Sex, *n* (%)	Women	98 (22.5)
Current age (years), median (IQR)		45.50 (36.25–53.0)
BMI (kg/m^2^), median (IQR)		25.30 (22.87–28.68)
Marital status, *n* (%)	Single	144 (33.0)
Marital status, *n* (%)	Cohabiting/Married	167 (38.3)
Marital status, *n* (%)	Divorced/Separated	117 (26.8)
Marital status, *n* (%)	Widowed	8 (1.8)
Employment status, *n* (%)	Employed	113 (25.9)
Employment status, *n* (%)	On sick leave	66 (15.1)
Employment status, *n* (%)	Unemployed	199 (45.6)
Employment status, *n* (%)	Retired	48 (11.0)
Employment status, *n* (%)	Homemaker	10 (2.3)
Educational level, *n* (%)	Primary or less	148 (33.9)
Educational level, *n* (%)	Secondary	219 (50.2)
Educational level, *n* (%)	University	69 (15.8)
SUD diagnosis, *n* (%)	AUD	436 (100.0)
SUD diagnosis, *n* (%)	Cocaine use disorder	206 (47.2)
SUD diagnosis, *n* (%)	Cannabis use disorder	88 (20.2)
SUD diagnosis, *n* (%)	Sedative use disorder	33 (7.6)
SUD diagnosis, *n* (%)	Stimulant use disorder	17 (3.9)
SUD diagnosis, *n* (%)	Opioid use disorder	19 (4.4)
SUD diagnosis, *n* (%)	Comorbid SUD	255 (58.5)
Nicotine use, *n* (%)	Yes	308 (70.6)
Nicotine use, *n* (%)	No	128 (29.4)
AUD diagnostic timeframe, *n* (%)	Before the past year	109 (25.0)
AUD diagnostic timeframe, *n* (%)	Past year only	15 (3.4)
AUD diagnostic timeframe, *n* (%)	Both before and during the past year	312 (71.6)
Age at alcohol initiation (years), median (IQR)		16.00 (14.00–18.00)
Age at AUD diagnosis (years), median (IQR)		25.00 (19.00–34.00)
AUD severity criteria, median (IQR)		8.00 (6.00–9.00)
Alcohol abstinence (days), median (IQR)		60.00 (29.25–180.00)
Stage of alcohol initiation, *n* (%)	Adolescence (10–21 years)	409 (93.8)
Stage of alcohol initiation, *n* (%)	Adulthood (>21 years)	27 (6.2)

Values are *n* (%) or median (IQR). AUD, alcohol use disorder; BMI, body mass index; IQR, interquartile range; SUD, substance use disorder.

**Table 2:** Clinical and alcohol-related characteristics according to developmental stage of alcohol initiation.

Domain	Characteristic	Early Adolescence (10–13 Years)	Middle Adolescence (14–16 Years)	Late Adolescence (17–21 Years)	*p*-Value
Participants, *n*		85	182	142	—
Sex, *n* (%)	Men	72 (84.7)	141 (77.5)	109 (76.8)	0.315 ^a^
Sex, *n* (%)	Women	13 (15.3)	41 (22.5)	33 (23.2)	0.315 ^a^
Current age (years), median (IQR)		45.00 (35.00–53.00)	43.00 (34.00–53.00)	47.00 (38.00–53.00)	0.143 ^b^
Criminal record, *n* (%)	Yes	34 (40.0)	70 (38.5)	33 (23.2)	0.006 ^a^
Criminal record, *n* (%)	No	51 (60.0)	112 (61.5)	109 (76.8)	0.006 ^a^
SUD diagnosis, *n* (%)	AUD	85 (100.0)	182 (100.0)	142 (100.0)	-
SUD diagnosis, *n* (%)	Cannabis use disorder	25 (29.4)	45 (24.7)	14 (9.9)	<0.001 ^a^
SUD diagnosis, *n* (%)	Cocaine use disorder	46 (54.1)	94 (51.6)	55 (38.7)	0.028 ^a^
SUD diagnosis, *n* (%)	Stimulant use disorder	7 (8.2)	9 (4.9)	1 (0.7)	0.018 ^a^
SUD diagnosis, *n* (%)	Sedative use disorder	7 (8.2)	15 (8.2)	11 (7.7)	0.985 ^a^
SUD diagnosis, *n* (%)	Opioid use disorder	3 (3.5)	13 (7.1)	1 (0.7)	0.016 ^a^
Comorbid SUD(s), *n* (%)	Yes	57 (67.1)	118 (64.8)	67 (47.2)	0.001 ^a^
Comorbid SUD(s), *n* (%)	No	28 (32.9)	64 (35.2)	75 (52.8)	0.001 ^a^
Number of SUD diagnoses, median (IQR)		2 (1–3)	2 (1–3)	1 (1–2)	<0.001 ^b^
Age at AUD diagnosis (years), median (IQR)		19.00 (16.00–25.00)	23.50 (18.00–30.00)	30.50 (22.00–38.75)	<0.001 ^b^
AUD severity criteria, median (IQR)		8.00 (6.00–10.00)	8.00 (6.00–9.00)	7.00 (5.00–9.00)	0.003 ^b^
Alcohol abstinence (days), median (IQR)		60.00 (14.00–180.00)	60.00 (17.25–180.00)	90.00 (30.00–180.00)	0.298 ^b^
Tobacco use, *n* (%)		65 (76.5)	138 (75.8)	100 (70.4)	0.461 ^a^
Cigarettes per day, median (IQR)		10.00 (5.00–20.00)	15.00 (2.25–20.00)	15.00 (1.00–20.00)	0.657 ^b^

Values are *n* (%) or median (IQR). ^a^ Chi-square test or Fisher’s exact test. ^b^ Kruskal–Wallis test. AUD, alcohol use disorder; IQR, interquartile range; SUD, substance use disorder.

**Table 3:** Cannabis- and cocaine-related characteristics according to developmental stage of alcohol initiation.

Domain	Characteristic	Early Adolescence (10–13 Years)	Middle Adolescence (14–16 Years)	Late Adolescence (17–21 Years)	*p*-Value
Cannabis use disorder, *n* (%)	Yes	25 (29.4)	45 (24.7)	14 (9.9)	<0.001 ^a^
Cannabis use disorder, *n* (%)	No	60 (70.6)	137 (75.3)	128 (90.1)	<0.001 ^a^
Age at cannabis use onset (years), median (IQR)		13.50 (12.00–15.75)	14.00 (11.00–16.50)	16.00 (12.00–17.00)	0.457 ^b^
Age at cannabis use disorder diagnosis (years), median (IQR)		15.00 (13.00–17.25)	17.00 (14.50–19.00)	16.50 (13.50–25.75)	0.427 ^b^
Cannabis use disorder severity criteria, median (IQR)		6.00 (3.00–8.00)	4.00 (2.00–8.00)	2.00 (2.00–5.25)	0.013 ^b^
Cannabis abstinence (days), median (IQR)		75.00 (28.75–365.00)	90.00 (30.00–365.00)	45.00 (13.25–365.00)	0.752 ^b^
Cocaine use disorder, *n* (%)	Yes	46 (54.1)	94 (51.6)	55 (38.7)	0.028 ^a^
Cocaine use disorder, *n* (%)	No	39 (45.9)	88 (48.4)	87 (61.3)	0.028 ^a^
Age at cocaine use onset (years), median (IQR)		15.00 (14.00–17.50)	18.00 (16.00–20.00)	22.00 (19.00–28.00)	<0.001 ^b^
Age at cocaine use disorder diagnosis (years), median (IQR)		20.00 (17.00–27.75)	23.00 (19.00–28.00)	28.00 (23.25–35.00)	<0.001 ^b^
Cocaine use disorder severity criteria, median (IQR)		9.00 (7.00–10.00)	9.00 (7.00–10.00)	7.00 (5.00–10.00)	0.012 ^b^
Cocaine abstinence (days), median (IQR)		26.00 (5.00–60.00)	85.00 (11.25–333.75)	35.00 (7.75–90.00)	0.027 ^b^

Values are *n* (%) or median (IQR). Ages, severity criteria, and abstinence duration were calculated only among participants with the corresponding disorder. ^a^ Chi-square test or Fisher’s exact test. ^b^ Kruskal–Wallis test. IQR, interquartile range.

**Table 4:** Ordinal logistic regression models for earlier developmental stage of alcohol initiation.

Model	Variable	OR for Earlier Initiation	95% CI	*p*-Value
Model 1: Including age at AUD diagnosis	Current age	1.02	1.00–1.05	0.036
Model 1: Including age at AUD diagnosis	Age at AUD diagnosis	0.92	0.90–0.94	<0.001
Model 1: Including age at AUD diagnosis	Childhood conduct disorder	3.42	1.50–7.76	0.003
Model 1: Including age at AUD diagnosis	Mood disorder	2.13	1.41–3.21	<0.001
Model 2: Excluding age at AUD diagnosis	Childhood conduct disorder	2.84	1.26–6.40	0.012
Model 2: Excluding age at AUD diagnosis	Mood disorder	1.62	1.10–2.40	0.016

The outcome was ordered so that OR > 1 indicates greater odds of belonging to an earlier initiation stage. For brevity, this table reports the statistically significant predictors from the fully adjusted models; the complete prespecified model, with all candidate predictors, model fit, and the test of parallel lines, is provided in Table S5. AUD, alcohol use disorder; CI, confidence interval; OR, odds ratio.

## Data Availability

The de-identified data supporting the findings are available from the corresponding author upon reasonable request and subject to ethics and data-protection restrictions.

## Supplementary Materials

The following supporting information can be downloaded at: https://www.mdpi.com/article/10.3390/medsci14050535/s1, Table S1, psychiatric comorbidity and treatment-related characteristics according to stage of alcohol use onset during adolescence; Table S2, medical comorbidities according to stage of alcohol use onset during adolescence; Table S3, specific psychiatric diagnoses assessed with the PRISM interview according to stage of alcohol use onset during adolescence; Table S4, ordered trend analyses across developmental stages of alcohol initiation; Table S5, ordinal logistic regression models for an earlier developmental stage of alcohol initiation: complete set of prespecified predictors; Table S6, latent class analysis: model-fit comparison of 1- through 5-class solutions.

## Abbreviations

The following abbreviations are used in this manuscript:

ADHD	Attention-deficit/hyperactivity disorder
AIC	Akaike information criterion
aBIC	Sample-size-adjusted Bayesian information criterion
AUD	Alcohol use disorder
BIC	Bayesian information criterion
BMI	Body mass index
CI	Confidence interval
CPD	Provincial Center for Drug Dependence
df	Degrees of freedom
DSM-5	Diagnostic and Statistical Manual of Mental Disorders, Fifth Edition
FDR	False discovery rate
IQR	Interquartile range
LCA	Latent class analysis
OR	Odds ratio
PEIBA	Andalusian Biomedical Research Ethics Portal
PRISM	Psychiatric Research Interview for Substance and Mental Disorders
SUD	Substance use disorder
