# Supplementary material for: Developmental Timing of Alcohol Initiation as a Marker of Adult Clinical Complexity in Alcohol Use Disorder: A Cross-Sectional Study
Source: Med Sci (Basel). 2026 Aug 31;14(5):535. doi: 10.3390/medsci14050535 (PMC13609073; doi:10.3390/medsci14050535)
Supplement: Supplementary file 1 [file medsci-14-00535-s001.zip › Table_S1.pdf]

Table S1. Psychiatric comorbidity and treatment-related characteristics according to stage of alcohol use onset during adolescence.

Variable		Early adolescence (10–13 years) *n* = 85	Middle adolescence (14–16 years) *n* = 182	Late adolescence (17–21 years) *n* = 142	*p*-Value
**Psychiatric comorbidity, *n* (%)**	Yes	58 (68.2)	123 (67.6)	81 (57.0)	0.097 [a]
	No	27 (31.8)	59 (32.4)	61 (43.0)	
**Type of psychiatric comorbidity, *n* (%)**	Mood disorder	48 (56.5)	82 (45.1)	55 (38.7)	0.034 [a]
	Anxiety disorder	20 (23.5)	52 (28.6)	38 (26.8)	0.687 [a]
	Psychotic disorder	9 (10.6)	15 (8.2)	10 (7.0)	0.644 [a]
	ADHD	21 (24.7)	39 (21.4)	23 (16.2)	0.264 [a]
	Personality disorder	22 (25.9)	40 (22.0)	26 (18.3)	0.397 [a]
**Psychotropic medication, *n* (%)**	Yes	67 (78.8)	137 (75.3)	106 (74.6)	0.758 [a]
	No	18 (21.2)	45 (24.7)	36 (25.4)	
**Type of psychotropic medication, *n* (%)**	Antidepressants	45 (52.9)	76 (41.8)	64 (45.1)	0.231 [a]
	Anxiolytics	38 (44.7)	81 (44.5)	57 (40.1)	0.690 [a]
	Antipsychotics	12 (14.1)	23 (12.6)	8 (5.6)	0.060 [a]
	Anticraving medication	40 (47.1)	82 (45.1)	81 (57.0)	0.088 [a]
**Psychological or substance use treatment/support, *n* (%)**	None	7 (8.2)	23 (12.6)	14 (9.8)	0.002 [a]
	Outpatient care	30 (35.3)	97 (53.3)	87 (61.3)	
	Hospitalization	9 (10.6)	8 (4.4)	7 (4.9)	
	Both outpatient care and hospitalization	39 (45.9)	54 (29.7)	34 (23.9)	
**Therapeutic community treatment, *n* (%)**	Yes	25 (29.4)	32 (17.6)	17 (11.9)	0.004 [a]
	No	60 (70.6)	150 (82.4)	125 (88.1)	
**Mutual support group participation, *n* (%)**	Yes	43 (50.6)	51(28.1)	39 (27.5)	<0.001 [a]
	No	42 (49.4)	131 (71.9)	103 (72.5)	

[(a)] *p*-Values using the chi-square test or Fisher's exact test. Values are presented as *n* (%). Abbreviations: ADHD, attention-deficit/hyperactivity disorder.
