# Supplementary material for: Developmental Timing of Alcohol Initiation as a Marker of Adult Clinical Complexity in Alcohol Use Disorder: A Cross-Sectional Study
Source: Med Sci (Basel). 2026 Aug 31;14(5):535. doi: 10.3390/medsci14050535 (PMC13609073; doi:10.3390/medsci14050535)
Supplement: Supplementary file 1 [file medsci-14-00535-s001.zip › Table_S2.pdf]

Table S2. Medical comorbidities according to stage of alcohol use onset during adolescence.

Variable		Early adolescence (10–13 years) *n* = 85	Middle adolescence (14–16 years) *n* = 182	Late adolescence (17–21 years) *n* = 142	*p*-Value
**Current age (years), median (IQR)**		45.00 (35.00–53.00)	43.00 (34.00–53.00)	47.00 (38.00–53.00)	0.143 [b]
**Any medical illness, *n* (%)**	Yes	41 (48.2)	74 (40.7)	60 (42.3)	0.501 [a]
	No	44 (51.8)	108 (59.3)	82 (57.7)	
**Type of medical illness, *n* (%)**	Neurological	3 (3.5)	15 (8.2)	7 (4.9)	0.250 [a]
	Musculoskeletal	8 (9.4)	9 (4.9)	2 (1.4)	0.021 [a]
	Endocrine	5 (5.9)	14 (7.7)	11 (7.7)	0.846 [a]
	Gastrointestinal	18 (21.2)	30 (16.5)	30 (21.1)	0.491 [a]
	Cardiovascular	8 (9.4)	18 (9.9)	14 (9.9)	0.992 [a]
**Multiple medical conditions, *n* (%)**		7 (8.2)	14 (7.7)	4 (2.8)	0.126 [a]

[(a)] *p*-Values using the chi-square test or Fisher's exact test; [(b)] *p*-Values using the Kruskal-Wallis test. Values are presented as *n* (%) or median (IQR). Abbreviations: IQR, interquartile range.
