# Supplementary material for: Developmental Timing of Alcohol Initiation as a Marker of Adult Clinical Complexity in Alcohol Use Disorder: A Cross-Sectional Study
Source: Med Sci (Basel). 2026 Aug 31;14(5):535. doi: 10.3390/medsci14050535 (PMC13609073; doi:10.3390/medsci14050535)
Supplement: Supplementary file 1 [file medsci-14-00535-s001.zip › Table_S3.pdf]

**Table S3**. Specific psychiatric diagnoses assessed with the PRISM interview according to stage of alcohol use onset during adolescence.

| Variable | Early adolescence (10–13 years) *n* = 85 | Middle adolescence (14–16 years) *n* = 182 | Late adolescence (17–21 years) *n* = 142 | *p*-Value |
|---|---|---|---|---|
| **Mood disorders, *n* (%)** | | | | |
| **Major depressive disorder** | 43 (50.6) | 78 (42.9) | 55 (38.7) | 0.217 [a] |
| **Dysthymia** | 1 (1.2) | 5 (2.7) | 2 (1.4) | 0.581 [a] |
| **Manic episode** | 4 (4.7) | 3 (1.6) | 1 (0.7) | 0.102 [a] |
| **Hypomanic episode** | 4 (4.7) | 1 (0.5) | 3 (2.1) | 0.093 [a] |
| **Cyclothymia** | 4 (4.7) | 1 (0.5) | 1 (0.7) | 0.020 [a] |
| **Anxiety disorders, *n* (%)** | | | | |
| **Specific phobia** | 2 (2.4) | 1 (0.5) | 1 (0.7) | 0.347 [a] |
| **Social phobia** | 2 (2.4) | 2 (1.1) | 5 (3.5) | 0.335 [a] |
| **Panic attacks** | 11 (12.9) | 11 (6.0) | 11 (7.7) | 0.157 [a] |
| **Agoraphobia** | - | 2 (1.1) | - | 0.286 [a] |
| **Generalized anxiety** | 5 (5.9) | 15 (8.2) | 8 (5.6) | 0.604 [a] |
| **Obsessive-compulsive disorder** | 1 (1.2) | 4 (2.2) | 1 (0.7) | 0.528 [a] |
| **Post-traumatic stress disorder** | 5 (5.9) | 22 (12.1) | 10 (7.0) | 0.152 [a] |
| **Psychotic disorders, *n* (%)** | | | | |
| **Schizophrenia** | - | 2 (1.1) | - | 0.286 [a] |
| **Schizoaffective disorder** | - | - | - | - |
| **Schizophreniform disorder** | - | 1 (0.5) | - | 0.535 [a] |
| **Delusional disorder** | - | 2 (1.1) | - | 0.286 [a] |
| **Psychotic disorder not otherwise specified** | 3 (3.5) | 4 (2.2) | 3 (2.1) | 0.767 [a] |
| **Brief psychotic disorder** | 6 (7.1) | 5 (2.7) | 3 (2.1) | 0.111 [a] |
| **Eating disorders, *n* (%)** | | | | |
| **Anorexia nervosa** | 1 (1.2) | 1 (0.5) | 2 (1.4) | 0.719 [a] |
| **Bulimia nervosa** | - | 7 (3.8) | 2 (1.4) | 0.099 [a] |
| **Personality disorders, *n* (%)** | | | | |
| **Childhood conduct disorder** | 18 (21.2) | 17 (9.3) | 6 (4.2) | <0.001 [a] |
| **Antisocial personality disorder** | 14 (16.5) | 21 (11.5) | 7 (4.9) | 0.016 [a] |
| **Borderline personality disorder** | 13 (15.3) | 26 (14.3) | 22 (15.5) | 0.949 [a] |
| **Neurodevelopmental disorders, *n* (%)** | | | | |
| **ADHD** | 21 (24.7) | 39 (21.4) | 23 (16.2) | 0.264 [a] |

[(a)] *p*-Values using the chi-square test or Fisher's exact test. Values are presented as *n* (%). Abbreviations: ADHD, attention-deficit/hyperactivity disorder.
