# Supplementary material for: Developmental Timing of Alcohol Initiation as a Marker of Adult Clinical Complexity in Alcohol Use Disorder: A Cross-Sectional Study
Source: Med Sci (Basel). 2026 Aug 31;14(5):535. doi: 10.3390/medsci14050535 (PMC13609073; doi:10.3390/medsci14050535)
Supplement: Supplementary file 1 [file medsci-14-00535-s001.zip › Table_S4.pdf]

Table S4. Ordered trend analyses across developmental stages of alcohol initiation.

Variable	Early adolescence (10–13 years) *n* = 85	Middle adolescence (14–16 years) *n* = 182	Late adolescence (17–21 years) *n* = 142	Raw *p*-value for trend	FDR-adjusted *p*-value
**Childhood conduct disorder, (%)**	21.2	9.3	4.2	<0.001	<0.001
**Cannabis use disorder, (%)**	29.4	24.7	9.9	<0.001	<0.001
**Comorbid non-alcohol SUD, (%)**	67.1	64.8	47.2	0.001	0.003
**Mutual support group participation, (%)**	50.6	28.1	27.5	0.001	0.003
**Therapeutic community treatment, (%)**	29.4	17.6	11.9	0.001	0.003
**Criminal record, (%)**	40.0	38.5	23.2	0.004	0.007
**Antisocial personality disorder, (%)**	16.5	11.5	4.9	0.004	0.007
**Mood disorder, (%)**	56.5	45.1	38.7	0.011	0.015
**Cocaine use disorder, (%)**	54.1	51.6	38.7	0.014	0.017

Raw trend *p*-values were obtained using two-sided Cochran–Armitage trend tests (equivalent linear-by-linear association tests) for binary variables. Values are percentages within each developmental stage of alcohol initiation. FDR-adjusted *p*-values were calculated with the Benjamini–Hochberg procedure across all 11 prespecified binary trend tests. The table displays the nine indicators that remained significant after correction; psychiatric comorbidity (raw p = 0.057; FDR-adjusted p = 0.063) and childhood ADHD (raw p = 0.108; FDR-adjusted p = 0.108) were included in the correction family but are not displayed.

Abbreviations: ADHD, attention-deficit/hyperactivity disorder; FDR, false discovery rate; SUD, substance use disorder.
