# Supplementary material for: Developmental Timing of Alcohol Initiation as a Marker of Adult Clinical Complexity in Alcohol Use Disorder: A Cross-Sectional Study
Source: Med Sci (Basel). 2026 Aug 31;14(5):535. doi: 10.3390/medsci14050535 (PMC13609073; doi:10.3390/medsci14050535)
Supplement: Supplementary file 1 [file medsci-14-00535-s001.zip › Table_S5.pdf]

Table S5. Ordinal logistic regression models for an earlier developmental stage of alcohol initiation: complete set of prespecified predictors.

Variable	β	SE	OR	95% CI	*p*-Value
Model 1 — including age at AUD diagnosis (n = 404)					
Current age	0.023	0.011	1.02	1.00–1.05	0.036
Sex (female)	0.043	0.250	1.04	0.64–1.70	0.864
Criminal record	0.122	0.229	1.13	0.72–1.77	0.592
No. of SUD diagnoses	0.098	0.237	1.10	0.69–1.76	0.680
Cocaine use disorder	0.067	0.344	1.07	0.55–2.10	0.845
Cannabis use disorder	0.443	0.370	1.56	0.75–3.21	0.231
No. of AUD severity criteria	−0.017	0.044	0.98	0.90–1.07	0.706
Mood disorder	0.756	0.210	2.13	1.41–3.21	<0.001
Childhood conduct disorder	1.228	0.418	3.42	1.50–7.76	0.003
Antisocial personality disorder	−0.436	0.423	0.65	0.28–1.48	0.302
Age at AUD diagnosis	−0.081	0.012	0.92	0.90–0.94	<0.001
Model 2 — excluding age at AUD diagnosis (n = 409)					
Current age	−0.004	0.010	1.00	0.98–1.02	0.708
Sex (female)	−0.194	0.238	0.82	0.52–1.31	0.415
Criminal record	0.172	0.224	1.19	0.77–1.84	0.442
No. of SUD diagnoses	0.161	0.230	1.17	0.75–1.84	0.485
Cocaine use disorder	−0.008	0.332	0.99	0.52–1.90	0.981
Cannabis use disorder	0.532	0.356	1.70	0.85–3.42	0.136
No. of AUD severity criteria	0.056	0.041	1.06	0.98–1.14	0.169
Mood disorder	0.483	0.200	1.62	1.10–2.40	0.016
Childhood conduct disorder	1.045	0.414	2.84	1.26–6.40	0.012
Antisocial personality disorder	−0.165	0.415	0.85	0.38–1.91	0.691

Both models included all prespecified candidate predictors simultaneously in a cumulative-logit proportional-odds model. The outcome was ordered so that an OR greater than 1 indicates higher odds of belonging to an earlier initiation stage (early > middle > late adolescence). Reference categories were male sex and absence of the listed binary clinical condition; ORs for continuous predictors correspond to a one-unit increase. Wald 95%CIs and two-sided *p*-values are reported. Model 1 included age at AUD diagnosis; Model 2 excluded it because of its conceptual and temporal proximity to age at alcohol initiation. Analytic samples reflect listwise deletion of missing data.

Model fit. Model 1: likelihood-ratio $\chi^2 = 91.1$, df = 11, $p < 0.001$; McFadden pseudo-$R^2 = 0.108$; Nagelkerke pseudo-$R^2 = 0.231$; −2 log-likelihood = 753.6. Model 2: likelihood-ratio $\chi^2 = 42.3$, df = 10, $p < 0.001$; McFadden pseudo-$R^2 = 0.049$; Nagelkerke pseudo-$R^2 = 0.112$; −2 log-likelihood = 815.2.

Test of parallel lines (proportional-odds assumption). Model 1: $\chi^2 = 9.2$, df = 11, $p = 0.608$; Model 2: $\chi^2 = 10.1$, df = 10, $p = 0.432$. The non-significant results provided no evidence that the proportional-odds assumption was violated.

Abbreviations: AUD, alcohol use disorder; CI, confidence interval; df, degrees of freedom; OR, odds ratio; SE, standard error; SUD, substance use disorder; β, regression coefficient on the logit scale.
