# Supplementary material for: Developmental Timing of Alcohol Initiation as a Marker of Adult Clinical Complexity in Alcohol Use Disorder: A Cross-Sectional Study
Source: Med Sci (Basel). 2026 Aug 31;14(5):535. doi: 10.3390/medsci14050535 (PMC13609073; doi:10.3390/medsci14050535)
Supplement: Supplementary file 1 [file medsci-14-00535-s001.zip › Table_S6.pdf]

Table S6. Latent class analysis: model-fit comparison of 1- through 5-class solutions.

No. of classes	Log-likelihood	Param.	AIC	BIC	aBIC	Entropy	Smallest class, *n* (%)	Conv.
**1**	−1955.55	9	3929.1	3965.2	3936.6	—	408 (100.0)	Yes
**2**	−1843.40	19	3724.8	3801.0	3740.7	0.811	62 (15.2)	Yes
**3**	−1818.70	29	3695.4	3811.7	3719.7	0.675	33 (8.1)	Yes
**4**	−1805.90	39	3689.8	3846.2	3722.5	0.688	36 (8.8)	Yes
**5**	−1795.95	49	3689.9	3886.5	3731.0	0.721	36 (8.8)	Yes

Bernoulli latent class models were estimated using nine binary clinical indicators (criminal record, cocaine use disorder, cannabis use disorder, mood disorder, childhood conduct disorder, antisocial personality disorder, childhood ADHD, therapeutic community treatment, and mutual-support group participation) among the 408 participants with complete data for all indicators. The composite comorbid non-alcohol SUD indicator was excluded to avoid redundancy. Fit indices favored different solutions: BIC was lowest for two classes, aBIC for three classes, and AIC for four classes; therefore, no single class structure was unequivocally supported. The two-class solution was retained for primary descriptive presentation because it minimized BIC, had the highest entropy among the multi-class solutions, and provided a parsimonious and clinically interpretable summary. The three-class solution was also considered plausible and was examined as a sensitivity model, as described in the main text. Lower AIC, BIC, and aBIC values indicate better fit, whereas higher entropy indicates clearer classification; the optimal value for each criterion is shown in bold. The aBIC was calculated as $-2 \log L + k \ln[(n + 2)/24]$, with $n = 408$ and k equal to the number of estimated parameters. Smallest-class counts and percentages are based on maximum-posterior-probability assignment. Developmental stage of alcohol initiation was not used to construct the classes.
Abbreviations: ADHD, attention-deficit/hyperactivity disorder; AIC, Akaike information criterion; aBIC, sample-size-adjusted Bayesian information criterion; BIC, Bayesian information criterion; Conv., convergence; Param., number of estimated parameters; SUD, substance use disorder.
